# The Low Voices: Quality, Ethics, and Reach of Qualitative Data* **

**DOI:** 10.17533/udea.iee.v40n1e02

**Published:** 2022-03-17

**Authors:** Carmen de la Cuesta-Benjumea

**Affiliations:** 1 . Nurse, PhD. Full Professor, Universidad de Alicante, Spain. Email: ccuesta@ua.es Universidad de Alicante Universidad de Alicante Spain ccuesta@ua.es

## Introduction

*The low voices… these are the voices of children, the women who speak to themselves, the emigrants, the dead, the animals... The voices of those who do not want to dominate and feed themselves on words and tales. (Manuel Rivas)*

**Figure 1:**
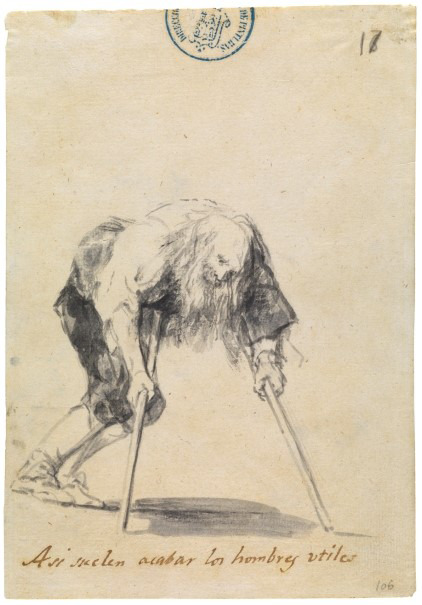
Goya. This is how useful men often end up. 1808-14. Notebook C page 17

**Figure 2:**
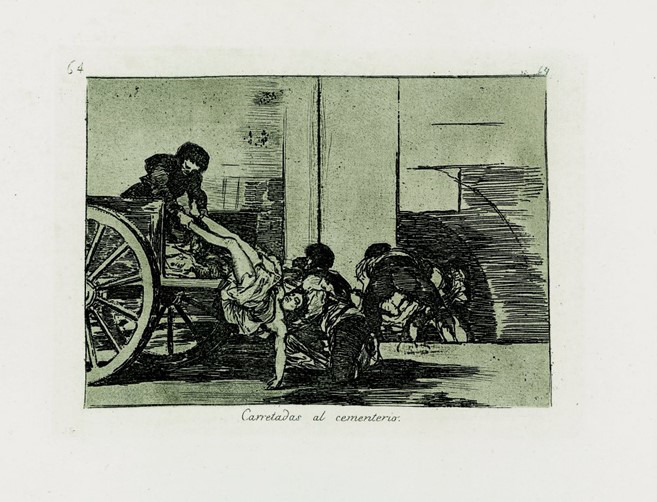
A and B. Goya: The disasters of war. Drawings made between 1810 and 1815

It may be stated that qualitative texts harbour a set of voices that, as if they were drawings, seek to sensitize and,ultimately- transform those who listen to them. These voices come from participants in studies, from ourselves, researchers, and from others who have preceded us and who speak to us from the literature. Our research studies set off from participant’s lived experience and, when interpreted, express the universal in the particular, as in the drawings by Goya who, by using images he took from the streets, expressed the pain and loneliness of people (Figure 1) and denounced events that occurred during the war, which over two-hundred years later still shudder and overwhelm us (Figure 2). Thus, similarly, carefully chosen and wisely combined in research reports, they, the low voices, will move those who read them into situations and experiences evoking potentially transformative images.

Carlos Castilla del Pino, Spanish psychiatrist during the 1950s reports in his memoires that: “his life was changed when he saw the resignation and humiliation of the families of the sick who came walking from the villages and slept on a bench in the street, overwhelmed by a disease they did not understand, which hurt them and they did not know how to fix”.(1) How is it possible “to see” resignation and humiliation? How does a health professional gain awareness of the suffering, disorientation, and sorrow of people? I believe that in order “to see” the subjective experience, it is necessary to have listened to it first.

Therefore, among all the voices that make up our research studies, this paper focused on the voices of the participants, the low voices who do not wish to dominate and who convey to us experiences of resignation, personal growth, or resistance and precisely for this reason, they cause a dent. Technically, this work is about qualitative data. I will first examine its nature; something to which little attention has been paid in the literature, then I will address the issue of obtaining qualitative data. I will end by exploring the reach of qualitative data in health care. I understand the importance of those working in health care to be sensitive, like Castilla del Pino, to see what is not visible to the naked eye. Health systems require compassionate individuals, people sensitive to suffering and willing to alleviate it. I am convinced that this sensitivity occurs when listening to the low voices, that is, when reading some studies that contain authentic qualitative data. I sustain that without quality data, it is not possible for a study to fulfil its transformative objective, that without ethically obtained data there is no legitimacy of the study and, hence, the study is questionable. Quality and ethics are two requisites for the transforming potential of qualitative research to be expressed. As qualitative health researchers, transformation in health care is our goal.

## The nature of qualitative data: their quality

In my opinion, the nature of qualitative data has been taken for granted and the definition that qualitative data is “that which is not numerical” has prevailed. Hence, I see it necessary to explore its nature, given that the quality of a study begins with its data.(2) Qualitative data must reflect the true essence of qualitative research, which is that of gathering the voice and perspective of whomever participates of an experience, Charmaz(3) illustrates it in the following text:

“Cristina suffers from chronic diseases and from their spirally increasing consequences. Her physical stress, frustration, and anger for the life she lives, her sadness, shame and uncertainty, all of which cause her suffering. Cristina talks a bit of the pain, and a lot of how difficult her life is made by the disability and the lack of money” (p.374)

In qualitative health research, we study how health-disease situations affect daily the lives of people knowing that what we capture are representations of a world, the subjective world, that cannot be captured directly.(4) Qualitative data are experiences interpreted and symbolized through words, thereby, they are mediated. In English, data obtained in research studies are denominated “raw”; in Spanish, we call them gross, but qualitative data are neither raw or gross. They are rich in a sense of abundance rather than of gastronomy.

Rich data are descriptive, detailed, and subjective. They present feelings, situations and events that are experienced first-hand by participants of the study in given situations, that is, qualitative data are specific. Let us see an example. Laura, during an interview, talks about her disease:

“This disease really has serious consequences for me, to say: I am already limited, I can’t have more children”, and maybe I would not have had more, but it has taken away the possibility of having them. It is silly, you think about it later, but yes, it's like that”.

As a matter of fact, the best way to express the quality of qualitative data is through the concept of richness. Knowing that we seek rich data, it provides us with a compass when, as researchers, we go out into the field to meet with people. Although we do not know exactly what it is we are looking for, but we must be clear about the aspect of that quality qualitative data has. Otherwise, we could end up gathering irrelevant, abstract and scattered information.

Rich data of quality must in addition be focused and complete. Richness is, thus, in terms of variety related to an issue, phenomenon or experience. Through rich data, we talk about something, not about everything. The data just quoted says little by itself. to be meaningful, these data should be linked to other data to become a category, theme or concept. In the above example, data are part of a category named “Not being the same person one used to be” that emerged in a study on the experience of living with chronic kidney disease. With this comment, we are now moving to the issue of data being intertwined with analysis and, thus, setting apart from the idea that qualitative data is simply the information that a tape recorder, a field diary or a document collects without trouble.

**Figure 3:**
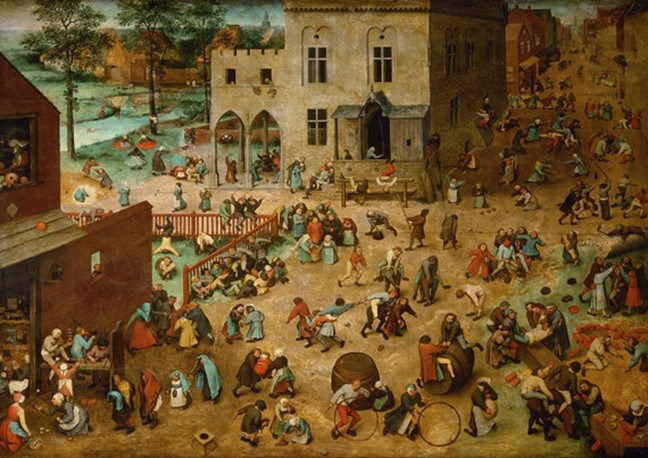
Pieter Bruegel the Elder, Children’s games (1560).

**Figure 4:**
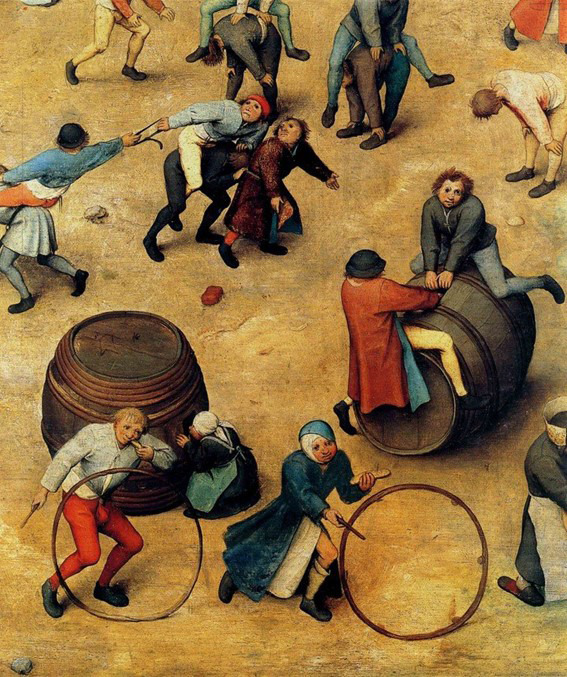
Pieter Bruegel the Elder, Children’s games (1560) Detail.

**Figure 5:**
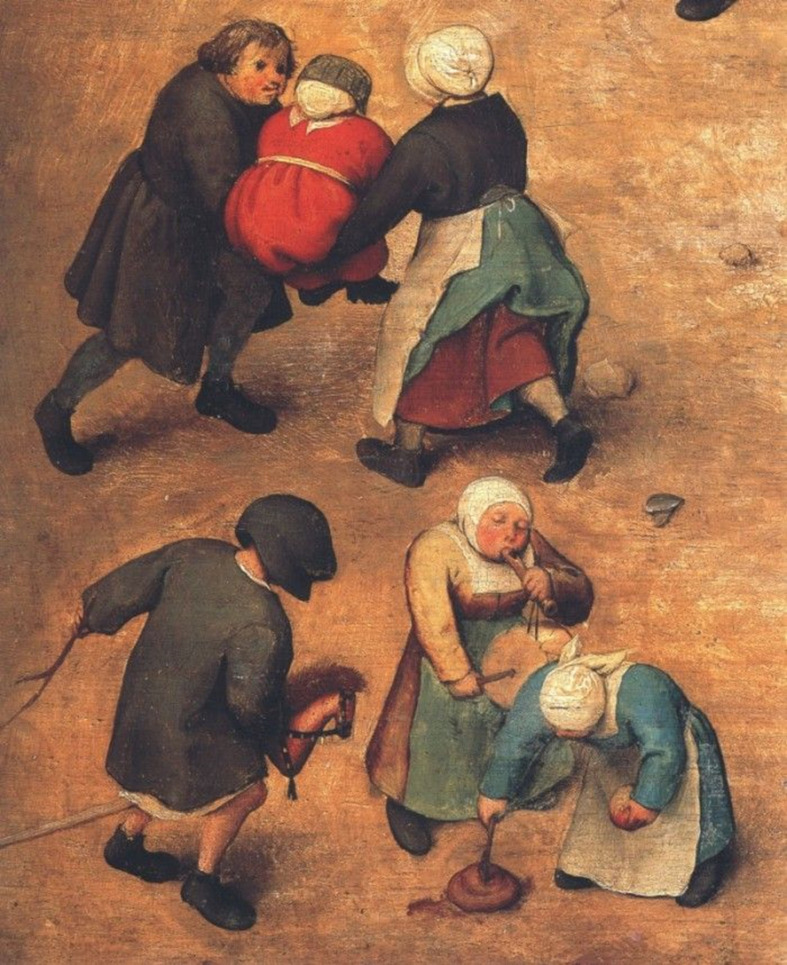
Pieter Bruegel the Elder, Children’s games (1560) Detail.

**Figure 6:**
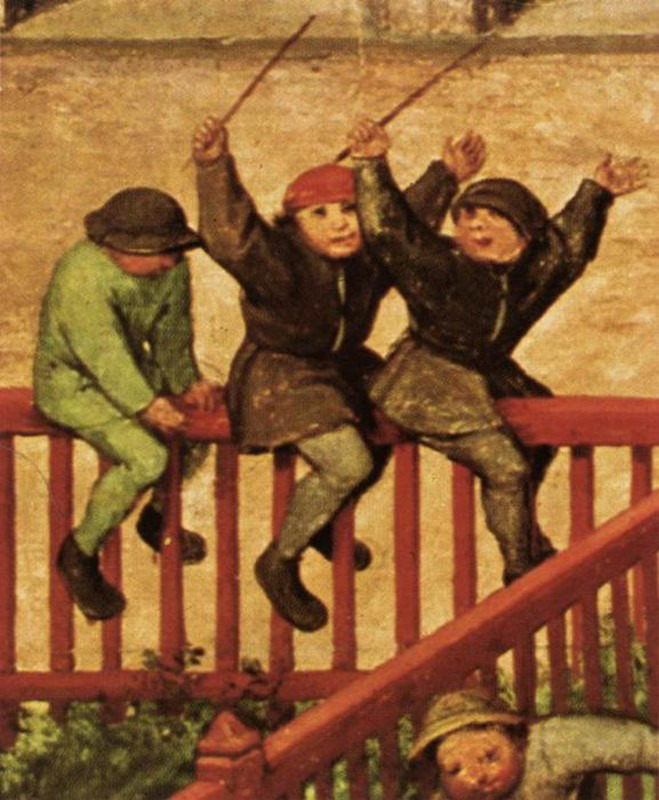
Pieter Bruegel the Elder, Children’s games (1560) Detail.

To explain the concepts of focused data and complete data, I will use a scene from everyday life in 1560 represented in the work by Flemish painter, Brueghel, titled “Children’s games”. In the same way that words trigger images, images trigger words and feelings. Thus, when contemplating this work for the first time, it can overwhelm us by the number of characters, details, information (Figure 3). In a closer look, we realise that, although varied, it deals with something specific, that it is focused on a theme and that is presented in complete manner, but not exhaustively. Thus, for example, we can identify diverse types of games (Figure 4), which vary the number of participants and where adults also participate (Figure 5). The images show children playing in Europe in the 16^th^ century, as well as depict the joy of playing (Figure 3 and Figure 6).

Focusing the data technically entails analysing it as we obtain it. Psychologically, focusing implies overcoming the fear of losing something important and giving up the idea of being exhaustive, ideas inherited from positivism. I know that giving up is not easy as it implies getting rid of some data that has been worked on hard and which we sense as valuable. But we must choose a path, take a route, or commit to an idea, we do not give up for nothing, but rather, to make the study feasible. If we do not do that, we will find ourselves overwhelmed or drowned in a mass of data, lost in details. We must, therefore, resist the temptation to say little about a lot.

The truth is, we gather more data than what we need. A good strategy to use these data, is to consider setting them aside for another study or just using them to complete and validate the topic on which the study has been focused. Thereby, we do not betray the study participants nor silence their voices; two moral issues that can unsettle us.

Relating to focusing, a question that students repeatedly ask is: How to choose, from all the possibilities that data present, the relevant issue or core experience? The answer is that whatever we choose, it is provisional and in order to choose what is substantial to participant’s experience, we connect with their experience. In this endeavour, will come to our aid our theoretical and social sensitivity, our intuition and having spent sufficient time in the field. After hard work, what is substantial in our data will come to us.

Once focused, data must be described completely. Complete description refers to accounting for thesignificant and greatest variety around the issue chosen, theme or phenomenon. With words we construct images, therefore, in this description we must not overwhelm with unnecessary detail.; We must capture the significant without dwelling on the details, show what is is substantial to the experience and describe it completely. A complete description, in my opinion, transmits an idea that captures an essential and subjective meaning, with no holes in it.

In technical terms, complete data refer to saturation, the means that tells us that the data is sufficient to provide an adequate account of an experience or phenomenon. Nevertheless, there is a tendency to confuse saturation with information redundancy, which is the repetition of information. This occurs when the researcher asks the same questions to all the study participants; of course, in this case, a point will come where the answers will be repeated without adding anything relevant. Quantity and depth are two concepts that, regarding qualitative data, cannot be separated. As a matter of fact, the greatest threat to the quality of research is that of scarce and superficial data; these will lead to simplistic and an obvious analysis (5) that will evoke nothing, nor transform anyone.

Focused and complete data make categories, themes, or concepts transcend the specific and connect us to with universal issues. Let us set an example, the category “protective governing ”(6) that emerged during a study on high-risk pregnancies; I have used this category to explain data from a study of people with chronic kidney disease and to understand the situation that a colleague whose child has functional diversity was sharing with me. When during our conversation I told her that what she was doing daily in relation with her child was “protective governing” , immediately this category readily echoed her experience.

In short, focused, and complete data are already interpreted data. We select from all the collected information, which is data for our study and as the study develops, these selections change. Somehow, we join the voices of the study participants. Low voices that have been constructed as they have been pondered and listened to. This is the subject of the following section.

## Obtaining data of quality

It is obvious that qualitative data are not collected, these are not a finished product ready to be removed and it is clear that its quality does not depend on technical procedures. Qualitative data are obtained, and their quality will depend on a process that is, to my understanding, a social process of relations and interactions. Wolcott, an insightful ethnographer, pointed out that “one seeks knowledge in the professional role of researcher, but prays for wisdom in the personal roles that make it possible” (p. 85).(7)

, Therefore, I consider it crucial to acknowledge that obtaining qualitative data of quality is linked to the researcher, to the research context, as well as to the study participants. Participants are not mere receptors of our questions, nor containers of experiences ready to be pulled out. The nature of the interactions with participants, the questions we ask during the field work, the instruments we use to obtain data and ethical issues will be matters that condition the quality of the data. 

So, obtaining qualitative data is the truly collaborative work that David Sudnow(8) illustrates in his study on the social organization of death:

I have tried to make friends in each of the hospital units to find out about certain reserved aspects of the wards. Sometimes I was treated with real enthusiasm and the staff also participated in my research. Many County [hospital] [physicians] interns, recently graduated and eager to show me their expertise in the world of biophysical facts, gave me lengthy lectures on the structure of the human body, completed with live observations in the wards. The interns [physicians] themselves, in their eagerness to be useful to me in this regard, have insisted more than once that I «touch here», «place your hand here», «feel this». (p. 14)

Therefore, we, researchers, obtain data through the relations we establish with the study participants. These are relationships of trust constructed during the very act of obtaining data. During these relations, the meaning of the experience is built and negotiated, nothing is imposed. Likewise, when obtaining data, as during any social encounter, we manage impressions, from this, the care we take in our aspect and in presenting ourselves to participants. We are aware that this will impact upon the relations and finally on obtaining data of quality. People talk to us because they like us or because they want to help us, nobody and nothing forces them to do that. Hence, during the field work, we adopt a style that invites to revealing information and to obtaining narratives of subjective depth where affective issues, such as empathy, understanding, care, and sensitivity play a key role on the type of data we obtain.(5)

The voices of the participants in our studies are low, intimate, emotional, and detailed. They are motivated by questions that shape these voices;(2) questions that imply a way of seeing the world and that can prevent or facilitate the emergence of subjective experience. For example, it is not the same to ask a person with functional diversity about their health problem as it is to ask her/him about the problems they face daily due to living in an environment not adapted to her/his functional diversity. As we can imagine, different people´s experiences will be constructed depending on how these questions are formulated. Truly, one of the challenges in obtaining qualitative data is being able to ask from the emic point of view before knowing it. Ethnographers are well aware of this dilemma and, therefore, claim that questions must first be discovered.(9) Hence, solely researchers’ empathys and their sensitivity will allow them to reach those deep levels of subjectivity and disclosure of facts, situations, and feelings.

Our first approaches to the experience to be revealed will be exploratory and, in the very process of obtaining the data, we will be like pulling a thread or following the trail of what seems significant to us in order to step-by-step get close to the subjective experience. Thus,the answers to our first questions will became new questions; but this is not done mechanically, rather, it requires researcher’s wonder and flexibility.(9) Wonder to discovering our assumptions and flexibility to follow the thread of a story or events without intruding . Within these interactions, it is where data are constructed with participants of our studies; so we must be attentive and be analytical during these interactions.(2) Our questions will shape the story being told, and the way we listen will make it possible. Thereby, it is already clear that the means to obtain qualitative data are instruments in the hands of the researcher; instruments do not have a life of their own. Indeed, the fact that we use a guide to conduct a qualitative interview does not make it qualitative, nor does the fact that the technique of content analysis of a text make it reveal meanings, nor does a field observation make it reveal cultural themes., A special ingredient must be added to the method, that is, a specific intention.(10) The habit does not a monk make.

Giventhat obtaining data is achieved through relationships of trust and affection, it isimbued by ethical issues, which, if not given due attention, can do harm and can invalidate a study. Here, reflexivity takes an ethical nature. When we are face to face with study participants , when we are listening to them, asking them questions or accompanying them in their "daily routine of small contingencies" as stated by Goffman(11) (p. 9), we verify that the approval of an ethics committee does not mean that study’s ethical issues have been overcome or resolved, as a large part of the ethical issues are emerging in qualitative studies. Thus, Lipson(12) talks about the dilemmas we face when, precisely because of the trust we have built, the study participants can place themselves at danger by providing us with some information, as happened to her in her study with refugees. Gastaldo and McKeever(13) also highlight the issue of how to undo the link with the participants at the end of the study. Although, as has been recognized by various researchers (2,14,15), it is not uncommon for this link to remain afterwards. Ours relations, being created in the field, are not disposable relations.

In turn, Smet *et al.,*(16) account for the multiple positions researchers can have with the study participants: that of researcher, of clinician, of a temporary family member, and of friend. This multiplicity blurs static notions of the researcher’s role and, with it, the ethical ambiguities and moral dilemmas that may arise in the research process.(16)

We must keep in mind that our questions can cause stress or painful memories to participants and we, researchers, must learn to stop on time; although participants allow us to enter their private lives, we must not overstep. Data is not obtained at all costs; its quality must also be ethical. When conveying data to written text, we must take care to present the voices of the study participants honourably, the voices we present in the research texts must not undermine their sense of dignity. Keeping study participants present during the writing is an ethical and moral position that should remain with us.

Qualitative research is a way of being and not of doing, that is, we do what we do because we think and feel in a certain way and this way must be ethical and not technical. Hence, today approaches to obtaining data must be fundamentally empathic, where researchers position themselves in favour of the people or groups they study, hoping to use the results to improve their living conditions and to promote social policies.(17) This parts from conventional approaches in which the objective of data collection was to extract as much data as possible from informants(17) or to gather objective information to be used in a neutral manner. From the moment we, qualitative researchers, are interested in the emic point of view, our studies disturb, challenge the status quo; they contain the seed of transformation. With this matter, ends this paper.

## Transformation: the reach of the data

At this point we may ask ourselves, why so much effort? It is precisely the reach of data that justifies it. We work so that, based on rich data, coming from the low voices, people's lives improve. Lina Masana(18), cultural anthropologist expresses it as follows:

“That [the people interviewed] explain to us as much as they can about their disease experience so that we can learn from it, analyse it and develop proposals that, in honour of applied anthropology, can be put into practice to improve the lives of those affected”. (p. 250)

Precisely, everything just said about the kind of data that we must obtain and about the process of obtaining it is, truly, a means to achieve a change that can happen from the very moment in which data are produced. It is well documented,(14) the therapeutic effect that interviews can have for the study participants -especially phenomenological interviews-, as well as their impact on us, researchers. Atkinson(5) states that being a witness, listening, understanding and accepting the life story of another person can be transformative. Data can “help, enable and even obligate researchers to look beyond their privileges” and to recognize where they come from.(19) 

Participatory action research and inclusive research, in which people with disabilities collaborate in the research project,(20) clearly demonstrates this transformative effect. To the extent to which we interact and empathize with our study subjects, we become sensitive to their lives, concerns, and desires. Once we, as researchers, have changed and documented what we learnt, this change can reach others. Indeed, our studies, like our data, must be genuine.

In turn, the sociology of health or of medicine has been documenting that health systems have become a sort of bio-medical bureaucracies that process patients with serious failures, such as obstetric violence, (21) racism,(22) and, in general, dehumanization. Recent qualitative studies point to the discrimination that from health services is practiced against vulnerable collectives, like the homeless,(23) people with intellectual disability,(20) immigrants or poor women who professionals see as unreliable and individuals with little credibility.(24) The deficient conditions of public health services with the irresponsible cutbacks of staff and resources have been exposed during this pandemic, as well as the devastating effect that these conditions have on the health care workers.

In this regard, what can we do? Truly qualitative data allow us to craft a scientific text that persuades readers and moves consciences, which transfers images upon which one cannot be indifferent. Low voices well-articulated and well-heard may not change the world, but do intensify awareness and, as the poet says, “a more intense awareness can act upon the circumstances”.(25) I believe the challenge for the 21^st^ century is to pay attention to the low voices and act accordingly.
